# Geometry and Microstructure Control of Remanufactured Metallic Parts by Cold Spray Additive Manufacturing

**DOI:** 10.3390/ma16134735

**Published:** 2023-06-30

**Authors:** Andrea Garfias, Rodolpho Vaz, Vicente Albaladejo-Fuentes, Javier Sánchez, Irene Garcia Cano

**Affiliations:** Centre de Projecció Tèrmica, Departament de Ciència del Materials i Química Física, Universitat de Barcelona, Martí i Franquès, 1, 08028 Barcelona, Spain; agarfias@cptub.eu (A.G.);

**Keywords:** additive manufacturing, cold spray, microstructure, annealing, characterization

## Abstract

Cold Spray Additive Manufacturing (CSAM) is a thermal spray technique that is typically used for the repair of metallic components. One of the challenges of CSAM is to improve the geometrical accuracy of the sprayed parts, along with overcoming the inferiority of the mechanical properties of the deposits by tailoring their microstructure with different deposition strategies. For this, Cu, Al, Ti, and Ti6Al4V substrates were reconstructed by two Cold Spray (CS) methods: Traditional (T) and a novel strategy, Metal Knitting (MK). The final geometry, microstructure, and mechanical properties of the reconstructed parts by these two methods were compared. Additionally, we investigated the effects of annealing on the microstructure of sprayed components and its influence on adhesion, resistance to erosion, and abrasive wear. The results indicate that annealing effectively reduces the microstructure defects of the remanufactured parts (up to 30% porosity reduction) and improves the adhesive strength (i.e., below 30 MPa for as-sprayed deposits, and up to 160 MPa for heat-treated Ti4Al4V deposits). Notably, the abrasive and erosive resistance of the Cu and Al annealed deposits sprayed by MK gave very similar results compared to their bulk counterparts, suggesting that it is an efficient method for the reconstruction of damaged parts.

## 1. Introduction

Included among the novel AM technologies by the ASTM F42—Additive Manufacturing Committee [1], Cold Spray (CS) is a process that can overcome some limitations of the most established techniques (Selective Laser Melting (SLM), Laser Metal Deposition (LMD), and Electron Beam Melting (EBM)) for the production of 3D metallic components. These methods consist of melting the feedstock material by a focused heat source (either a laser or an electron beam), which then is resolidified to form an object [2]. In some cases, this could represent a major drawback since these thermal methods tend to produce defects within the microstructure, such as undesired phase changes, oxidation, and the formation of residual stresses [3].

CS is widely accepted as an AM solid-state process based on accelerating a powder feedstock by flowing it through a convergent−divergent (de Laval) nozzle with a preheated, compressed, supersonic gas (N_2_ or H_2_) to high velocities (300–1200 m/s). When the accelerated powder is sprayed into a substrate with enough kinetic energy (surpassing the critical velocity), plastic deformation occurs at the point of impact, promoting the formation of a well-bonded coating [4].

In recent years, the potential of CS as an additive manufacturing process has been studied [3,5,6,7,8,9]. Cold Spray Additive Manufacturing (CSAM) is an emerging technology that offers some advantages compared to other AM methods, including high deposition rates, deposition of temperature-sensitive materials, and minimization of tensile residual stresses [10]. However, some studies have shown that the as-sprayed components present defects, including rough surfaces and micropores that may cause poor cohesion between particles due to the particle−particle bonding mechanism. Apart from that, CSAM is still restricted to those applications in which an accurate control of strict geometric tolerances is not mandatory. To overcome the limitations that CSAM has on the microstructural characteristics, critical strategies like the deposition path trajectory, optimization of the process parameters, postheat treatments, and postmachining processes are still under study [7,8,9,11,12,13,14].

The deposition strategies and robotic path in CSAM, along with the process variables, play an important role in the final characteristics of the sprayed deposits. Parameters such as the impact angle, transversal velocity of the robot, feedstock flow rate, number of layers, temperature, and pressure determine fundamental features of the sprayed parts, like the final shape, microstructure, cohesion between particles, and bonding with the substrate. 

**Figure 1:**
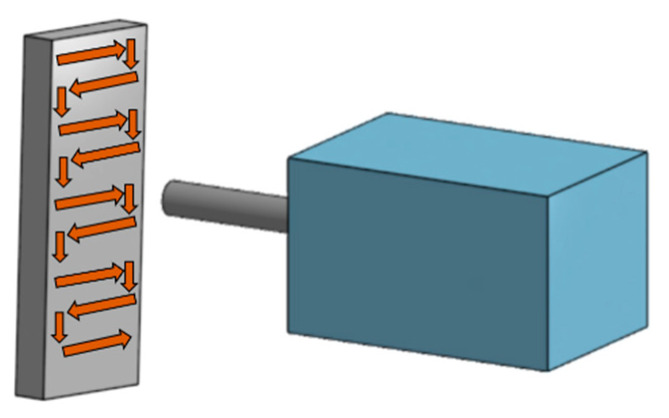
Cold spraying path of the traditional spraying method.

Traditionally, CS coatings are sprayed perpendicularly (at a constant angle of 90°) towards the substrate, following a straightforward path from one side to the other with a specific step, as shown in Figure 1. 

It is well known that a spraying angle of 90° increases the velocity component at which the particles impact the substrate; when this is greater than the critical velocity, the bonding between the particle−substrate improves, and higher deposition efficiencies are reached [15]. However, it is accepted that, when exiting the nozzle, the sprayed particles show a velocity profile approximated to a Gaussian distribution curve due to the friction of the outer particles with the walls of the walls nozzle, promoting higher deposition in the center of the jet spot [16]. With this traditional deposition strategy, the CSAM deposits tend to grow with a specific geometry, triangle, or pyramid shape [16]. In this sense, new robotic path trajectories are needed for more accurate control of the parts’ geometry. Some alternatives have been proposed in the literature [8,16,17,18]. For example, Wu et al. [16] developed a new building strategy that considers spraying at a deflection angle on the edges to build a straight wall. 

Most of these alternatives keep the traditional convection of retaining a normal substrate-gun angle for maximizing deposition efficiency; however, new interfaces and defects are normally generated in the build components apart from those specific to the CSAM process. In addition, these works are generally focused on the development of free-form building strategies for CSAM technology. However, not many studies consider a deposition strategy for the repair or remanufacture of a component in which the final geometry is restricted to the initial state of the specimen; for instance, thin-walled components.

**Figure 2:**
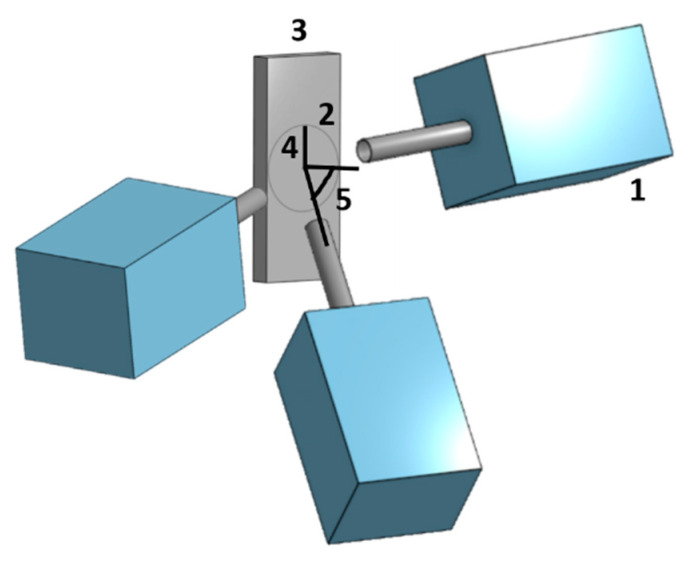
Knitting strategy scheme. (1) Cold Spray gun, (2) virtual cone path, (3) substrate, (4) radius, (5) angle.

The authors of this study have recently developed a new spraying methodology, Metal Knitting, based on a single movement of the spraying gun that allows spraying feedstock particles toward the substrate at a fixed angle (in this case, less than 90°) [19]. In this method, the nozzle is continuously moved following a circular path until one round is completed (forming a virtual cone), then the gun is moved one step to the side and the cycle is repeated as in a knitting movement. The strategy is visually explained in Figure 2. 

In contrast with traditional CS methodologies, Metal Knitting greatly advances CSAM as it allows control of the geometry of the deposit by growing straight walls with a repeatable strategy and a resolution falling within the range of millimeters. Additionally, with the MK strategy developed by the authors, thin-wall deposits can be generated at a faster rate, keeping high deposition efficiency, and even on top of predefined geometries. Nevertheless, spraying with an angle other than 90° cannot reduce the generation of micropores and interparticle boundaries within the microstructure, nor improve the low adhesion strength of the deposited material with the substrate [3]. This is why it still remains of vital importance to investigate new deposition strategies that allow the geometric and microstructure control of the deposits with the objective of adapting their final properties to the required performance of the components for their specific final application.

Thus, the aim of this study is to assess the microstructure and final shape of reconstructed Cu, Al, Ti, and Ti6Al4V substrates by the new Metal Knitting method and compare their final geometry, microstructure, and mechanical properties with the CS traditional strategy. Furthermore, the effect of annealing on the microstructure and performance of the deposits was evaluated in terms of adhesion and resistance to erosion and abrasive wear.

## 2. Materials and Methods

### 2.1. Feedstock Powder Characterization 

Commercial irregular and spherical powders, Al (Aluminum Industries Pvt Ltd., Hyderabad, India), Ti (CNPC Powder, Shanghai, China), Cu (SAFINA A.S., Vestec, Czech Republic), and Ti6Al4V (AP&C Powders Metallurgy, Montreal, Canada), respectively, were sprayed with Plasma Giken PCS100 (Saitana, Japan) equipment. The powder size distribution was analyzed with a laser diffraction particle sizing analyzer LS 13 320 Model Dry Powder System (Beckman Coulter, Brea, USA), while particle morphology was observed in a Phenom ProX Desktop scanning electron microscope (SEM) (Phenom-World BV, Eindhoven, The Netherlands).

### 2.2. Spraying Parameters

To compare the effect of the deposition strategy on the microstructure of the parts, all powders were sprayed with the Metal Knitting (MK) and traditional (T) methods using the spraying parameters shown in Table 1. All samples were deposited on the grit-blasted thin face (50 mm × 5 mm) of 20 mm × 50 mm × 5 mm substrates of the same material as the sprayed powder (Cu, Al, Ti, or Ti6Al4V). For the MK samples, transversal velocity, angle, and radius were equal for all samples. 

### 2.3. Heat-Thermal Treatment of CSAM Deposits

CSAM deposits generated by two different spraying strategies were heat-thermal treated in order to study the effect of an annealing postprocess in the deposits’ microstructure and the enhancement of particle−particle cohesion, mechanical properties, and wear behavior. All the samples were annealed in a tubular Carbolite Gero furnace (Sheffield, UK) under an argon atmosphere and cooled down in the furnace until room temperature. Annealing temperature and times used are included in Table 2. Annealed samples are labeled as (HT) throughout this study.

### 2.4. Characterization and Mechanical Properties

The microstructural characterization and the mechanical properties were determined for the samples produced by the two methodologies.

The metallographic preparation of all components was completed according to the standard ASTM E1920-03(2014). The cross-sectional surface of the polished samples was analyzed with the optical microscope (OM) Leica DMI5000M (Wetzlar, Germany). The software ImageJ (version 1.53e) was used to calculate the mean porosity value according to the standard ASTM E2109-01(2014).

The Vickers microhardness was tested following the standard ASTM E384-17 with a force of 0.2 and 1 kgf with Shimadzu HV-2/HMV-2T equipment (Kioto, Japan). Ten indentations were made and measured for each sample. 

The flattening ratio (*FR*) was calculated according to Equation (1), where *w* = width and *h* = height of the sprayed particles observed in the etched cross-sectional surface of the deposit [20]. The width was considered the widest part of the particle, indistinctive of the impact direction.

(1) $$FR=\frac{w}{h}$$

To analyze the phases of the deposits, the XRD equipment PANalytical X’Pert PRO MDP (Cambridge, UK) was used with radiation of Cu Kα (λ = 1.7903 Å) from 7 until 135° 2θ with a step size of 0.017°. 

**Figure 3:**
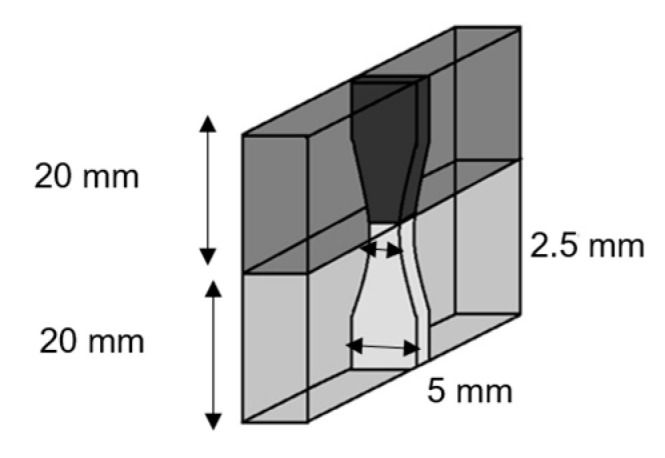
Tensile test specimens.

The deposit−substrate adhesion of the sprayed components with Metal Knitting (as-sprayed (AS) and heat-treated (HT)) was measured with tensile strength tests with equipment SERVOSIS MCH-102ME (Madrid, Spain). Three samples of each component (Figure 3) were subjected to a tensile test at a rate of 0.01 mm/s until cohesive or adhesive failure occurred. The failure mechanism of the tested samples was observed with a Phenom ProX Desktop SEM.

### 2.5. Resistance to Erosion and Abrasive Wear

The resistance to erosion of the thermally treated MK samples was tested following the standard ASTM G76-04 [21]. Alumina particles with a size of 50 µm were propelled at a constant feeding rate toward the samples’ surface at a normal angle. The erosion test was also performed on the substrates to compare the behavior of the additively produced samples and the bulk material. The average erosion value (mm^3^/g) was calculated by dividing the erosion rate (weight loss of the sample in time) by the abrasive flow rate and the density of the material.

The resistance to abrasive wear was calculated using the Rubber Wheel method following the standard ASTM G65-16e1 [22] with homemade equipment CM4 OL-2000. The test was performed at a velocity of 139 rpm with a force of 20 N, a wheel diameter of 22.9 cm, and Ottawa silica sand as an abrasive agent.

## 3. Results and Discussion

### 3.1. Feedstock Powder Characterization

**Figure 4:**
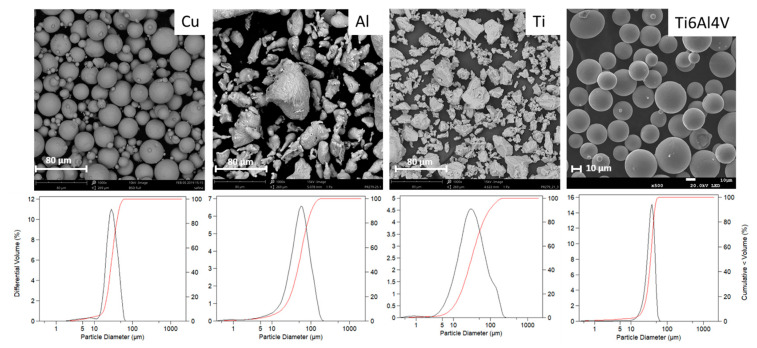
(**top**) SEM micrographs of the sprayed powders and (**bottom**) particle size distribution graphs.

Feedstock characteristics such as material, shape, and size distribution determine in great part the spraying parameters needed to obtain an optimal microstructure of the produced part. Table 3 and Figure 4 show the mean particle size, as well as SEM micrographs and size distribution curves of the powders. 

These results show that all powders exhibited a size distribution with a mean particle size between 30 and 55 µm. The selection of the powders was made so that Cu and Ti6Al4V were spherical, while Al and Ti were irregular. As pointed out by Vaz et al. [19] the characteristics of the deposits are influenced more by the nature of the material than by powder morphology. The selection of two different powder morphologies will allow us to conclude if this statement is still valid when the deposition process is adapted to a nonnormal deposition strategy.

### 3.2. Geometry Analysis of the Reconstructed Metallic Parts

**Figure 5:**
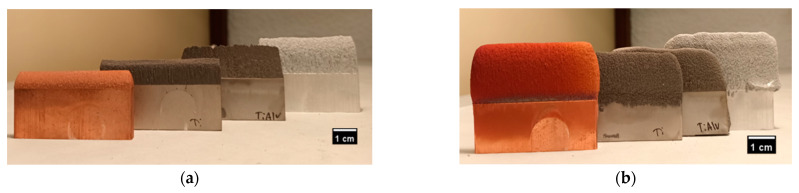

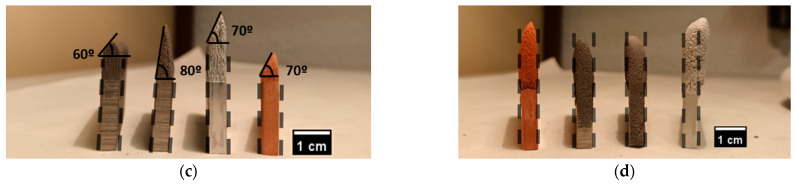
Images of the as-sprayed samples produced by (**a**,**b**) Traditional and (**c**,**d**) Metal Knitting strategies.

To investigate the possibility of remanufacturing straight elements by CS, Cu, Al, Ti, and Ti6Al4V, parts were sprayed by Traditional (T) and Metal Knitting (MK) strategies into the thin face (50 mm × 5 mm) of 20 mm × 50 mm × 5 mm substrates. The resulting parts of both methods are shown in Figure 5. It can be observed that the Traditional samples were only able to grow a short height without starting to generate an inclination in the sidewalls, producing a pyramid-shape deposit whose angle was dependent on the material. This is attributed to the profile velocities of the particles when they exit the nozzle during spraying; particles in the core of the jet spot tend to reach higher velocities, promoting a higher deposition in the center compared to the periphery [23]. The height limitation and wall inclination could represent a restriction in applications such as the remanufacture of thin-walled structures by traditional CS methods; therefore, new deposition strategies and robot path trajectories need to be studied. Wu et al. [16] proposed a deposition approach that involves spraying at a deflection angle on the edges of the pyramid-shaped deposits with the objective of obtaining freeform 3D objects with more precision. Additionally, as each material has its own inclination angle, a profile study for every material is necessary when using this strategy. On top of that, spraying at an angle different than normal directly onto a previously sprayed deposit could have a great influence on deposition efficiency, adhesion strength, and porosity of the deposits. 

Moreover, it is worth indicating that the T-Cu sample was surprisingly poorly adhered and detached from the substrate. After spraying, a clear crack appeared at the deposit−substrate interface that ended up with the built wall easily separated. Copper is known for being a good material for CS technology due to its high plastic deformability; nonetheless, this issue might have been associated with excess residual stresses accumulated in the deposit−substrate interface when a high copper thickness is intended to be deposited by CS. This phenomenon is also demonstrated in the study of Benenati et al. [8]. This result clearly indicates that, for some materials, the possibility of repairing this kind of geometry with conventional strategies is completely restricted and represents another reason to study new robot path trajectories.

**Figure 6:**
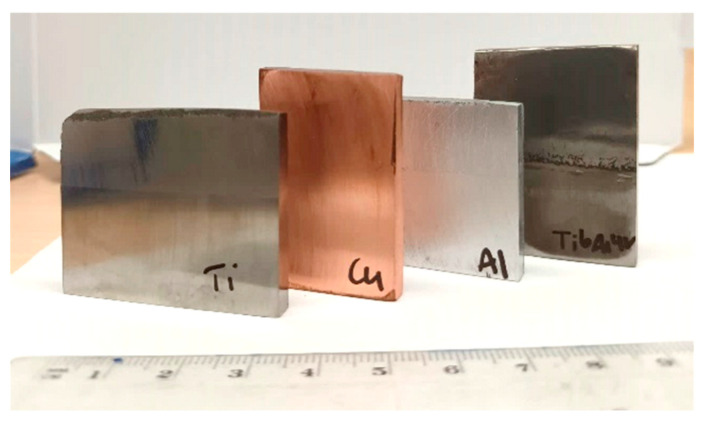
Postmachined Metal Knitting samples.

With the Metal Knitting strategy, the samples were able to grow vertically without an apparent limitation, producing straight walls without cracks or delamination areas in the interface between the part and the substrate. On top of that, the typical inclination is not observed because the nozzle is moved in a circular way, possibly hindering the effects of the velocity differences in the jet spot and promoting an even growth of the components. In terms of geometry, the MK method overcomes the limitations of the Traditional one, allowing more accurate control of the shape of the deposits. However, the samples showed a rough finish, and their shape still deviates from the precision that most final applications would require. To improve on this, the components need to be subjected to postmachining processes, as shown in Figure 6. It can be observed that the samples were well bonded at the interface and that an almost identical part was reconstructed on top of the thin wall of the substrate.

### 3.3. Characterization of the Microstructure and Physical Properties of the CS Deposits 

**Figure 7:**
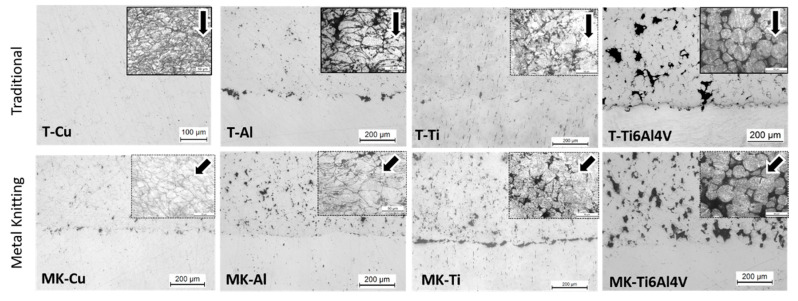
Polished and etched cross-section images (optical microscope) of Traditional (**top**) and Metal Knitting (**bottom**) samples. Lines indicate the spraying direction.

Along with the component’s geometry, the study of the microstructure characteristics is very important to understand further the effects that the spraying strategies may have on the final properties of the deposits. To investigate this, the polished and etched cross-section was observed in an optical microscope (OM). The surface characteristics and particle boundaries of the as-sprayed samples produced by both strategies are shown in Figure 7, while the flattening ratio of the particles along with the microstructural properties of the deposits, such as porosity or microhardness, are shown in Table 4. For a good comparison of results, in the case of the T-Cu sample, microstructure inspection and physical properties were analyzed in the detached copper deposit. 

In general, all the deposits produced following the traditional spraying strategy exhibited a very dense microstructure, with the exception of T-Ti6Al4V that showed a microstructure with a high porosity value of 14 ± 4%. T-Cu, T-Al and T-Ti showed the common lamellae microstructure of CS deposits composed of highly deformed particles. This occurred independently from the original powder particle morphologies; for instance, Al and Ti particles (irregular powders) showed flattening ratios of 2.4 ± 1.0 and 1.8 ± 0.5, respectively. However, for T-Ti6Al4V, high porosity can be attributed to the presence of voids heterogeneously distributed across the microstructure of the deposit. Despite the fact that the spraying parameters had been previously optimized, the T-Ti6Al4V particles mostly maintained their original shape with a flattening ratio of 1.3 ± 0.4. It is widely accepted that this material is hard to deposit by CS because of its low plastic deformability and high critical velocity needed for deposition (above 1000 m/s), sometimes not achievable with commercial CS equipment [4,24]. Therefore, many studies of CS Ti alloys with high porosity are found in the literature [25,26,27]. Li et al. [27] have collected CS Ti and Ti alloy porosities of several works, resulting in porosity values that range from <1% up to 20% depending on spraying parameters such as gas temperature, pressure, or propulsive gas. 

In contrast to the traditional strategy, the Metal Knitting method allowed us to deposit well-bonded deposits of all the materials tested, including copper. With MK, the deflection of the particle impact angle with the surface might lead to a reduction of accumulated residual stresses in the interface, which allows getting well-bonded thick deposits [8]. The microstructure of these deposits showed identical features to the Traditional deposits and was composed of homogeneously deformed and compacted particles. Figure 7 clearly shows in the microstructure of MK-Cu, MK- Al, and MK-Ti, the modification of the impact angle of the particles as these appeared deformed along the spraying direction. Because of the modification of the spraying angle to values lower than 90° during the whole MK process, the MK deposits showed higher porosity and interparticle voids than the deposits generated by the traditional strategy. However, this porosity seemed to be homogeneous, and no clear cracks or lines of continuous porosity were observed in the microstructure of the deposits. Comparing the T- and MK-Ti6Al4V deposits, a similar flattening ratio was found, but a much higher porosity resulted with the MK method (14 ± 4% vs. 21 ± 5%, respectively) pointing out that the modification of the spraying angle caused a lack of deformation at impact, and bonding in the interface was hindered [15]. The MK and the traditional method showed minor differences with respect to the microstructural characteristics. In contrast, MK allows the obtention of thicker deposits with more accurate control of the geometry. 

### 3.4. Effect of Heat-Thermal Treatment on the Microstructure of CS Deposits

It is widely known that the as-sprayed deposits by CS can have unfavorable mechanical properties compared to bulk materials, including decreased strength, ductility, electrical and thermal conductivity, and wear resistance. This can be attributed to defects inherent in the cold spray process. When particles are plastically deformed in the solid state and packed together, micropores and interparticle boundaries can emerge in the microstructure, particularly when the material is not sufficiently ductile or when impact velocity is low [3]. Furthermore, because the particles are arranged in a layered pattern, anisotropic behavior has been observed in CSAM deposits [28].

This still remains one of the challenges of CSAM; therefore, several works related to the enhancement of the microstructure of the CS deposits are found in the literature. According to research, heat treatments have proven to be among the most successful approaches for improving the microstructure of CSAM deposits, as they can relieve residual stresses through the application of heat, reduce the incidence of microstructural defects such as porosity and particle boundaries, and enhance the adhesion between particles [3]. The effects that heat treatments have on the enhancement of the microstructure of CS deposits can be found in a wide range of materials, including copper [29,30], aluminum [12,31], titanium and its alloys [4,13,26,32,33], stainless steel [34], and others. One heat treatment that has become a topic of interest in the field of CSAM is hot isostatic pressing (HIP), which subjects samples simultaneously to high temperatures and isostatic pressures. HIP can remove pores and microcracks from deposits. The existing literature on the subject is limited, and most studies have concentrated on hard materials that are difficult to deposit using a cold spray, such as titanium [26], Ti6Al4V [27], and Inconel 718 [16]. However, this posttreatment process is limited by its high costs, the limited size of the samples, and the long cycle time. Moreover, another alternative that has been widely studied is annealing, a straightforward postprocessing technique that also has a favorable effect on the microstructure of the sprayed components. It induces diffusion and recrystallization processes that reduce unwanted microstructural defects and softens the work-hardened deposits, increasing ductility and tensile strength but decreasing their hardness compared to their as-sprayed equivalents.

**Figure 8:**
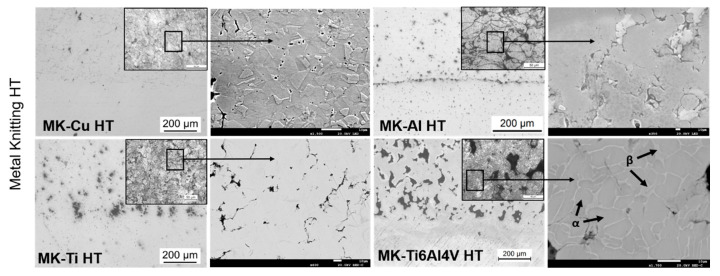
Polished and etched cross-section and images (optical microscope and SEM) of Metal Knitting heat-treated samples.

Therefore, to investigate the effect of posttreatment on the microstructure of the CSAM deposits, the Metal Knitting samples were subjected to an annealing process. The temperature and dwell time used for the annealing process of each material is indicated in the Materials and Methods section (Table 2). Only the sprayed parts by MK were used for this part of the study since it allowed the deposition of the whole variety of metals under study in this work. Additionally, the microstructures obtained with MK led to deposits with well-compacted particles, and it did not show a significant increase in porosity. Figure 8 shows OM and SEM micrographs of the polished heat-treated MK samples, while their microstructural properties are presented in Table 4. The OM images in Figure 8 show clear densification of the microstructure of the MK deposits when compared with the microstructures of the as-sprayed parts (Figure 7). In general, some differences are worth noting between as-sprayed and annealed samples. First, for all the materials, it is clearly seen that the particles’ microstructure changed because of a recrystallization process occurring for all the materials (see SEM image of MK-Cu HT deposit in Figure 8). In addition, the boundaries of the particles slightly disappeared after the annealing, indicating that in all the cases, the temperature was adequate for promoting metal diffusion through the particles’ boundaries. As a result, an increase in cohesion between particles and the enhancement of the mechanical properties of the deposits was expected. Finally, a reduction of the microstructure’s porosity is clearly observed for all the materials, which was confirmed by the calculation of the porosity percentage corresponding to each material and included in Table 4. In general, the voids generated in the interparticle boundaries and small pores disappeared, due to the recrystallization processes [12,13,14,32,35].

Despite the general reduction in porosity values achieved for all materials after the heat treatment, big pores and voids generated between particles during the deposition were still present. These defects were generated as a consequence of the insufficient plastic deformation of particles at impact. This issue was even more significant for the MK-Ti6Al4V deposits; its microstructure revealed the absence of a high number of deformed particles.

The microhardness of the Traditional and MK as-sprayed and MK heat-treated deposits was measured in order to analyze the modification of this property after annealing. Similar microhardness values were measured for as-sprayed deposits regardless of the deposition strategy used. This result agrees with the microstructure and porosity evaluation of the deposits mentioned above, which showed no clear differences in particle deformation between the two deposition strategies under study. The small variations in microhardness between T and MK deposits can be correlated to the different particle impact angles used in each strategy. A reduction in the impact angle of the particles is known to result in less plastic deformation, which has two main consequences: (i) lower work hardening of the particles and therefore, lower hardness of the deposit, and (ii) reduced particle−particle cohesion, leading to weaker mechanical performance. As mentioned above, MK samples were subjected to heat-thermal treatments to enhance deposit microstructure and mechanical behavior. After the annealing, two tendencies were observed in the microhardness depending on the material; on the one hand, in the case of MK-Cu HT, MK-Al HT, and MK-Ti HT, the microhardness values were maintained or slightly decreased, which is typical behavior of these materials after annealing. On the other hand, the microhardness of MK-Ti6Al4V HT presented an increase in this value, suggesting that the thermal treatment generated a decrease in porosity and enhanced the cohesion of particles, increasing the microhardness values [36] and promoting the appearance of β phases [32], as can be observed in Figure 8. To confirm the phase changes that sample MK-Ti6Al4V HT underwent during the annealing, an XRD analysis was done; the results showed an increase in the intensity of the peak-related β phase after the heat treatment. This XRD diffractogram is presented in the supplementary section (S1).

### 3.5. Adhesion Strength of the Reconstrued Part to the Substrate

**Figure 9:**
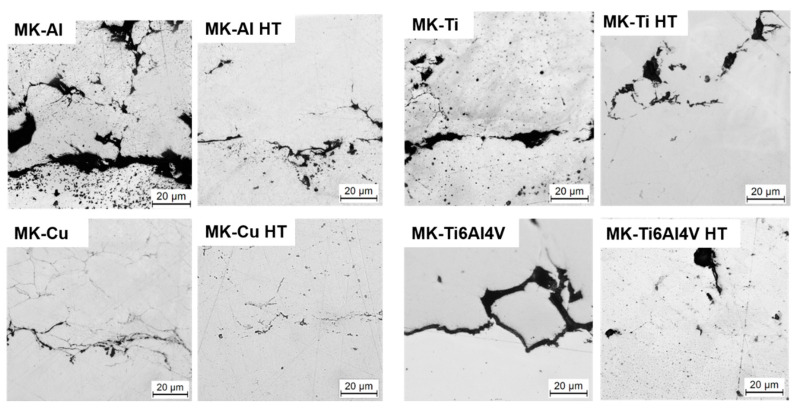
Optical microscope images of the deposits’ interface.

As mentioned before, it is widely accepted that spraying at an angle other than 90° hinders the bonding of the particles, promoting poor adhesion between the deposited particles and the substrate. Yin et al. [37] studied the effect of the spray angle on CS deposition. Briefly, they studied the behavior of single impact splats at different angles; for deposition at a 90° angle, the particles deform uniformly, while at lower angles, as is the case of Metal Knitting, a localized deformation is present, reducing the interfacial contact area. Since the use of these components is intended for the reconstruction of metallic parts, high adhesion values between particles and substrate are required. To achieve this, a smooth interface with intimate contact of the particles with the substrate is imperative [15]. Figure 9 displays the cross-sectional images of the interface of the MK as-sprayed and HT samples. The as-sprayed MK samples showed porous microstructures, mostly with a rough deposit−substrate interface, which may indicate poor bonding between the particles and the substrate caused by a lack of intimate contact due to the spraying angle. In general, it is considered that in the CGS process, two bonding mechanisms can be responsible for the particle−surface adhesion and particle−particle cohesion, mechanical interlocking, and metallurgic bonding. The extent of each bonding mechanism depends on the particle size, velocity, and impact angle [38]. The images of the interfaces of the as-sprayed MK deposits (Figure 9) clearly show that as a consequence of the modification of the impact angle, mechanical interlocking is the predominant bonding mechanism of these deposits to the substrate. An improvement in the adhesion of the deposits can be observed by looking at the interface of the HT MK samples which are smoother, without porosity, and more homogeneous than the MK- as-sprayed deposits. These images might indicate that metallurgical bonding occurred during the heat-treatment step of the MK deposits, suggesting an increased adhesion of the deposit compared to the as-sprayed samples [39,40]. 

**Figure 10:**
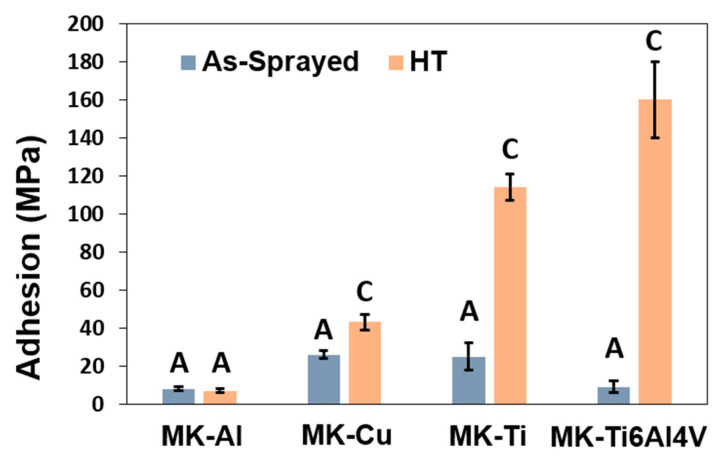
Tensile test results of as-sprayed and heat-treated (HT) specimens; (A) and (C) labels indicate adhesive or cohesive failure, respectively.

Furthermore, to investigate the adhesion of the Metal Knitting deposits in the as-sprayed and annealed state, a tensile test was performed (Figure 8). As expected from observing the surface characteristics in Figure 6 and interface microstructure in Figure 9, all MK as-sprayed samples showed adhesive failures at the deposit−substrate interface with values below 30 MPa, suggesting a poor bonding between the manufactured part and the substrate. Conversely, the MK HT samples (Cu, Ti, and Ti6Al4V) presented a cohesive failure with greater tensile strength values (43, 114, and 160 MPa, respectively) than their as-sprayed counterparts, which further confirms that annealing could be an adequate posttreatment process to increase the bonding between particles and substrate. It is worth indicating that the Al samples showed no change in the tensile strength value after the HT, probably because the annealing step does not promote enough diffusion across the splat boundaries to improve deposit−substrate adhesion, as can be observed in Figure 10 [12].

**Figure 11:**
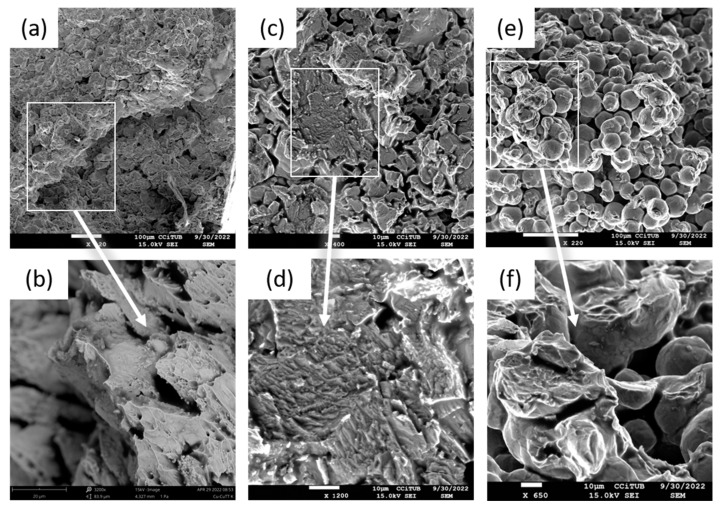
SEM micrographs of the specimens’ failure after tensile test of (**a**,**b**) MK-Cu HT, (**c**,**d**) MK-Ti HT, and (**e**,**f**) MK-Ti6Al4V HT.

The failure mechanism of the heat-treated MK samples that presented cohesive failure was observed by SEM, and the resulting images are presented in Figure 11. Overall, the samples showed signs of plastic deformation, indicative that the annealing strengthened the bonding between particles. The MK-Cu HT sample was highly deformed before failure, revealing a high deformation in the form of peaks in the specimen, while the MK-Ti HT and MK-Ti6Al4V samples showed a mixture of interparticle and intraparticle fracture propagation with the characteristic dimples that appear during plastic deformation in the intraparticle failure regions. All these results demonstrate that annealing is a good strategy to overcome the poor adhesion and cohesion obtained when spraying at an angle other than 90°.

### 3.6. Wear Resistance of Annealed CS Deposits to Erosive and Abrasive Conditions

One of the main challenges when reconstructing a damaged part for remanufacturing purposes is to achieve the required performance of the deposits for operating conditions; ideally, similar to that of the original component [41]. As has been previously discussed, CS is a particular repairing and remanufacturing technology since the bonding mechanism between smashed particles is attributed to two different mechanisms—mechanical interlocking and metallic microjunctions [39,40,42]. This fact makes it difficult to prepare deposits with very high particle−particle cohesion compared to other additive manufacturing processes. In order to guarantee that the new reconstructed part shows a similar behavior compared to the original material, the enhancement of particle cohesion is mandatory. In this sense, postspraying heat-thermal treatments are expected to improve not only the deposit microstructure and deposit adhesion to the substrate but internal cohesion of the rebuilt section by CS. In this study, we have carried out an evaluation of the resistance to erosive and abrasive wear for Metal Knitting HT samples. It is accepted that for thermally sprayed parts, the resistance to wear by hard particles is very sensitive to the quality of the deposits, that is, the presence of porosity, cracks, inclusions, oxides, etc. [7,28]. After annealing, it has been demonstrated that a significant improvement in MK deposit density and adhesion strength was achieved. The comparative analysis of erosion and abrasion wear rates of the annealed samples with a plate of the same material could bring light to the effect of this heat-thermal treatment on the particle cohesion of the MK deposits.

**Figure 12:**
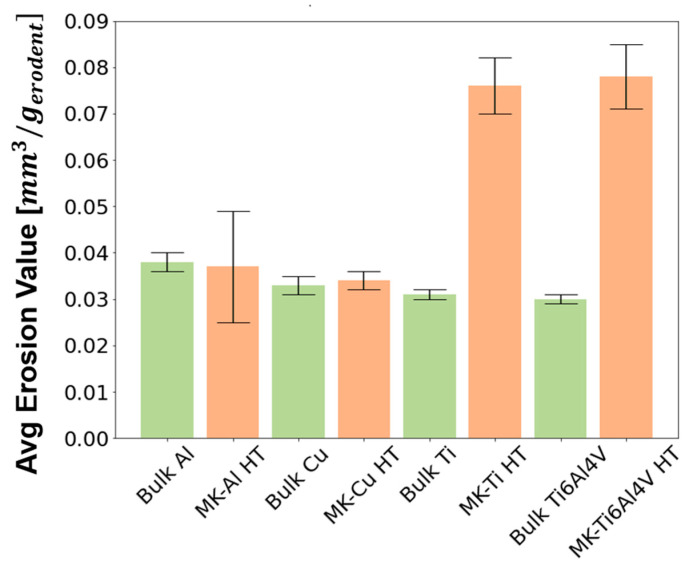
Average erosion value of the bulk and MK heat treated samples.

**Figure 13:**
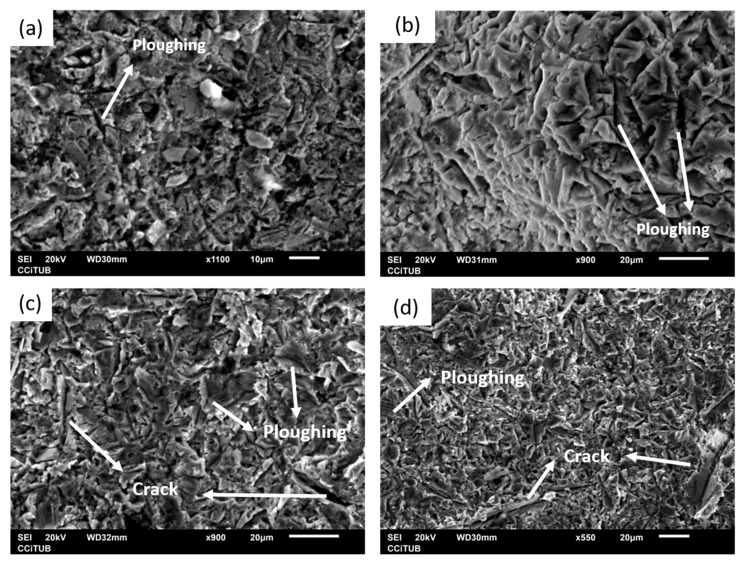
SEM micrographs of the erosion mechanism of (**a**) MK-Cu HT, (**b**) MK-Al HT, (**c**) MK-Ti HT, and (**d**) MK-Ti6Al4V HT.

As pointed out in the materials and methods section, for erosion tests, hard alumina particles with a size of 50 µm were propelled to the additively produced samples and the bulk plates with a normal incidence angle. Figure 12 shows the graph representing the average erosion value results of the tested samples. Here, it can be observed that the behavior of the bulk and deposited Cu and Al samples was very similar, as there were no important differences in their average erosion value. Lower resistance to erosion than their bulk counterpart resulted for the MK-Ti HT and MK-Ti6Al4V HT samples, which presented the highest volume loss in Table 5 (erosion values of 0.076 ± 0.006 and 0.078 ± 0.007, respectively). For a better understanding of the different results obtained for Cu and Al and Ti and Ti6Al4V pairs, SEM micrographs of the eroded surfaces were recorded for the MK samples after erosion testing. These images are shown in Figure 13. Two main wear mechanisms are generally involved in erosion damage—plastic deformation, and brittle fracture [43,44]. As could be expected for the case of ductile materials, the images of the eroded surface for MK-Cu HT and MK-Al HT showed clear signs of predominant erosion by plastic deformation of the surface. In this mechanism, erosive wear is associated with the detachment of small portions of metals by plastic rupture due to the impact of hard particles. Two types of deformation were identified in Figure 13a,b, which confirm this conclusion. In general, all the eroded surfaces show signs of deformation by ploughing but in addition, small and isolated signs of plastic failure by cutting can also be observed. This result can be perfectly explained considering that for these two materials, low porosity and high flattening ratios of the deposits were observed even for the as-sprayed samples. After annealing, the microstructure of the MK-Cu and MK-Al clearly showed a reduction in porosity and an improvement of cohesion between deposited particles due to intraparticle recrystallization processes. As a result of achieving high particle−particle cohesion, a similar erosion resistance to the bulk material was measured for MK-Cu HT and MK-Al HT coupons when hard abrasive particles collided with their surfaces. This result demonstrates that Metal Knitting, followed by a posttreatment process, is a successful method for the reconstruction of Cu and Al components while controlling the final geometry of the deposit with a single-movement and fast CS deposition strategy.

MK-Ti HT and MK-Ti6Al4V clearly showed a different behavior than the Cu and Al samples despite the fact that these are considered ductile materials too. Certainly, the SEM images of the eroded Ti and Ti6Al4V surfaces also showed sins of plastic deformation (mainly the ploughing type) as one of the mechanisms contributing to the wear of the samples; however, for these two materials clear and big cracks were additionally identified in the surface. These cracks revealed the presence of voids or big pores in the microstructure of the deposits even after annealing, which had been previously observed in the MO microstructure analysis after the heat-thermal treatment. Considering that Ti and its alloys are not brittle material, the identification of cracks on their eroded surface would be hardly attributed to the severe impact of hard particles. In contrast, crack patterns might indicate the easy propagation of an interparticle due to the low cohesion shown for these deposits. Extended collision of erosive materials will completely pull out the deposited particles, which in the end, justifies the high erosion value measured for MK-Ti HT and MK-Ti6Al4V HT samples [44,45].

**Figure 14:**
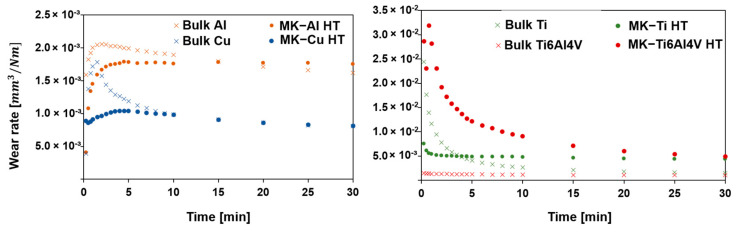
Wear rate of the bulk and MK heat-treated samples.

In agreement with the erosion tests, the wear resistance to abrasive conditions, by a Rubber Wheel test was performed on the sprayed samples and the bulk (used as a reference value). Figure 14 shows the plot of the wear rate throughout the experiment. Moreover, Table 5 presents the data values for the volume loss and wear rate (both measured at the end of the test). SEM images of the abraded surfaces are not shown since only signs of abrasion were observed. During the first 5 min, most samples showed a steep increase or decrease in the wear rate, followed by a stabilization of this value after this time. This behavior is possibly attributed to a rapid decrease of the superficial roughness and to the pull-out of the last layer of the deposited particles which are those showing the lowest particle−particle cohesion since they were not affected by the tamping effect of a new layer of particles. 

According to the work presented by Fu et al. [46], the abrasive wear resistance of materials is directly associated with properties such as Young’s modulus, toughness, hardness, and compactness. Again, the same trend as in the erosion resistance test can be observed. On the one hand, MK-Cu HT and MK-Al HT samples showed very similar resistance to abrasive wear compared to pure Cu and Al plates, respectively, confirming that Metal Knitting is a suitable technique for repairing components fabricated with these two materials. For the MK-Ti HT and MK-Ti6Al4V HT samples, a considerable difference in the wear rate and volume loss with their Ti and Ti6Al4V counterparts was observed. As revealed by the erosion experiments, the Rubber Wheel tests confirmed that for MK HT samples of Ti and its alloy, the annealing heat treatment carried out in this study did not improve the compactness of the particles to a degree where their behavior could be comparable with the bulk. Results obtained with Cu and Al coupons sprayed by Metal Knitting have demonstrated that postspraying thermal treatment is a good option for improving deposit quality and performance. Even though this specific annealing process did not allow enhancing the microstructure of the Ti and Ti6Al4V, the results of this study suggest that further heat treatments such as hot isostatic pressing (HIP) or spark plasma sintering (SPS) should be evaluated in the future [33]. Furthermore, future studies should consider the manufacturing of more complex shapes, along with the CS remanufacture of materials that are of interest in particular applications, for example, IN718 to reconstruct or repair damaged turbine blades. 

## 4. Conclusions

CSAM is an AM method with great benefits compared to other heat-focused techniques. It is still an emerging technology in which some challenges are yet to be studied. This work focused on improving the geometry control accuracy using a spraying strategy for remanufacturing metallic parts and tailoring the final microstructure of CSAM deposits with annealing heat treatments. Several findings can be concluded:

1. With Traditional CS strategies, the accumulation of residual stresses in the particle-substrate interface and the poor adhesion of the deposit might lead to the detachment of the reconstructed part during spraying, as in the case of T-Cu. Moreover, they are not suitable for the production of thick coatings with strict geometric tolerance, because as the deposits are sprayed, a pyramid-shaped part starts to grow. This normal deposition strategy does not allow the successful deposition of all kinds of materials.
2. Metal Knitting is an effective CSAM deposition strategy for the reconstruction of metallic parts compared with the Traditional method. With MK, straight walls of different materials with unlimited height and without detachment of the deposits can be obtained. However, the deposition angle reduces the plastic deformation of the particles, increasing porosity within the microstructure and hindering adhesion with the substrate.
3. Annealing was found to be an effective heat-treatment process to tailor the microstructure of the samples by improving the cohesion between particles and adhesion with the substrate, as well as reducing porosity.
4. The MK-Cu HT and MK-Al HT samples showed similar characteristics as the bulk materials in terms of mechanical properties and resistance to abrasive and erosive wear, suggesting that the manufacturing of these materials by CSAM MK followed by annealing could be comparable to other bulk production methods.
5. The porosity and low particle−particle cohesion observed in the microstructure of the MK-Ti and MK-Ti6Al4V samples were not significantly enhanced, even after the annealing treatment, which explains their low resistance to erosive and abrasive wear in comparison to the bulk. For these alloys showing high critical impact velocities, the results of this study suggest that a heat-thermal treatment is insufficient for the improvement of the deposit quality, and other densification strategies are required.

## Figures and Tables

**Table 1:** Feedstock powders and gas spraying parameters.

Feedstock Powder	Supplier	Pressure (bar)	Temperature (°C)	Standoff Distance (mm)	Deposition Strategy Label
Cu	Safina	30	700	30	Metal Knitting: MK
Al	Arasan	30	450	30	
Ti	CNPC	62	700	25	Traditional: T
Ti6Al4V	AP&C	65	1000	25	

**Table 2:** Heat-thermal treatment conditions for CSAM deposits.

Label	Material	Atmosphere	Temperature (°C)	Time
-HT	Cu	Argon	600	4 h
	Al		400	
	Ti		1000	
	Ti6Al4V		1000	

**Table 3:** Mean particle size of the sprayed powders.

Powder	Shape	Mean Particle Size (µm)	d10 (µm)	d90 (µm)
Cu	Spherical	29.7 ± 2.9	18.7 ± 2.6	43.2 ± 5.2
Al	Irregular	54.8 ± 4.5	19.4 ± 1.2	97.3 ± 4.9
Ti	Irregular	40.1 ± 2.8	9.7 ± 0.8	89.5 ± 6.4
Ti6Al4V	Spherical	30.7 ± 3.1	13.2 ± 8.6	45.2 ± 0.4

**Table 4:** Microstructural characterization and flattening ratio.

Feedstock	Traditional			Metal Knitting				
	As-Sprayed			As-Sprayed			Heat Treated	
	Flattening Ratio	Porosity (%)	Microhardness (HV0.2)	Flattening Ratio	Porosity (%)	Microhardness (HV0.2)	Porosity (%)	Microhardness (HV0.2)
Cu	2.2 ± 0.8	3 ± 2	91 ± 7	2.3 ± 0.6	6 ± 2	88 ± 10	4 ± 1	60 ± 8
Al	2.4 ± 1.0	3 ± 1	49 ± 8	2.7 ± 0.7	10 ± 4	50 ± 3	7 ± 2	31 ± 7
Ti	1.8 ± 0.5	7 ± 3	262 ± 43	1.7 ± 0.3	12 ± 3	199 ± 22	8 ± 2	203 ± 27
Ti6Al4V	1.3 ± 0.4	14 ± 4	236 ± 32	1.2 ± 0.3	21 ± 5	229 ± 31	16 ± 3	275 ± 25

**Table 5:** Resistance to erosion and abrasive wear results.

Sample	Erosion Resistance	Abrasive Wear Resistance	
	Average Erosion Value (mm^3^/g)	Wear Rate (mm^3^/Nm)	Volume Loss (mm^3^)
Bulk Al	0.038 ± 0.002	1.6 × 10^−3^	240
MK-Al HT	0.037 ± 0.012	1.8 × 10^−3^	259
Bulk Cu	0.033 ± 0.002	8.0 × 10^−4^	41
MK-Cu HT	0.034 ± 0.002	8.0 × 10^−4^	41
Bulk Ti	0.031 ± 0.001	1.5 × 10^−3^	76
MK-Ti HT	0.076 ± 0.006	4.4 × 10^−3^	222
Bulk Ti6Al4V	0.030 ± 0.001	1.0 × 10^−3^	53
MK-Ti6Al4V HT	0.078 ± 0.007	5.0 × 10^−3^	248

## Data Availability

The data presented in this study are available on request from the corresponding author.

## Supplementary Materials

The following supporting information can be downloaded at: https://www.mdpi.com/article/10.3390/ma16134735/s1, Figure S1: XRD results of (a) Ti6Al4V powder, (b) MK-Ti6Al4V, and (c) MK-Ti6Al4V HT.

## Nomenclature

AS	As-sprayed
CS	Cold spray
CSAM	Cold spray additive manufacturing
EBM	Electron Beam Melting
FR	Flattening Ratio
HT	Heat-treated
LMD	Laser Metal Deposition
MK	Metal Knitting
OM	Optical microscope
SEM	Scanning Electron Microscope
SLM	Selective Laser Melting
SPS	Spark plasma sintering
T	Traditional
HIP	Hot isostatic pressing
