# Supplementary material for: Geometry and Microstructure Control of Remanufactured Metallic Parts by Cold Spray Additive Manufacturing
Source: Materials (Basel). 2023 Jun 30;16(13):4735. doi: 10.3390/ma16134735 (PMC10342963; doi:10.3390/ma16134735)
Supplement: Supplementary file 1 [file materials-16-04735-s001.zip › materials-2443402-supplementary.pdf]

Supplementary material

Geometry and Microstructure Control of Remanufactured Metallic Parts by Cold Spray Additive Manufacturing

Andrea Garfias, Rodolpho Vaz, Vicente Albaladejo-Fuentes *, Javier Sánchez and Irene Garcia Cano

Centre de Projecció Tèrmica, Departament de Ciència del Materials i Química Física, Universitat de Barcelona, Martí i Franquès, 1, 08028 Barcelona, Spain; agarfias@cptub.eu (A.G.); rvaz@cptub.eu (R.V.); jsanchez@cptub.eu (J.S.); igcano@cptub.eu (I.G.C.)

* Correspondence: valbaladejo@cptub.eu

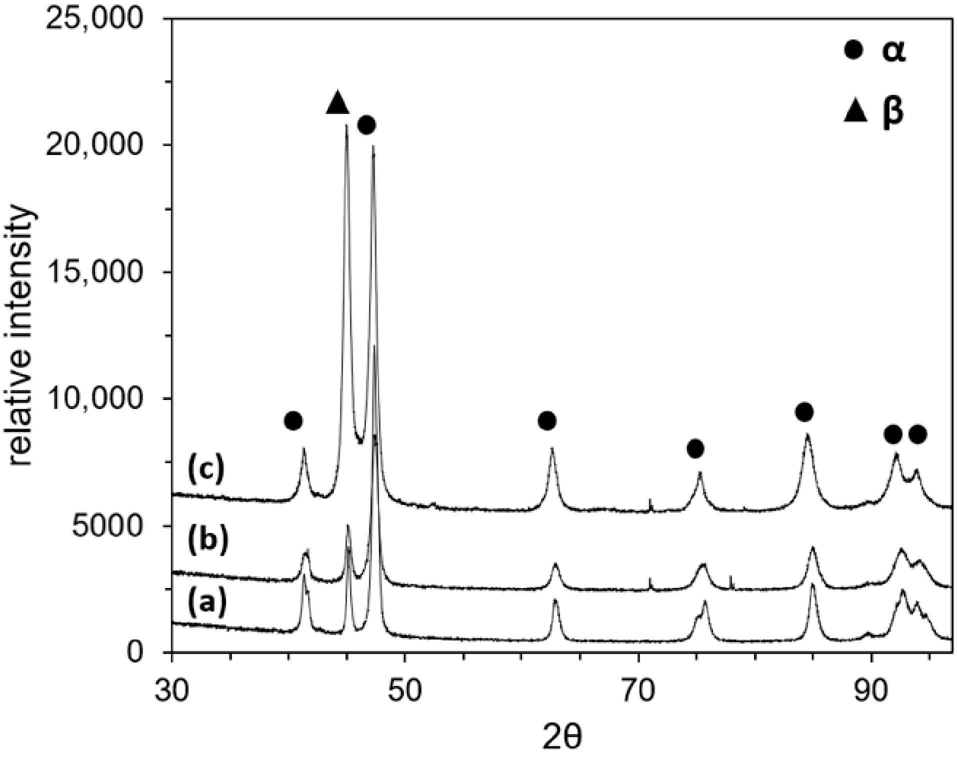

Figure S1. XRD results of (a) Ti6Al4V powder, (b) MK-Ti6Al4V, and (c) MK-Ti6Al4V HT
